# Prior Authorization, Quantity Limits, and Costs for Varenicline in Medicare

**DOI:** 10.1001/jamanetworkopen.2025.0008

**Published:** 2025-03-03

**Authors:** Changchuan Jiang, Ryan D. Nipp, Arthur S. Hong, Ya-Chen Tina Shih, Jiazhang Xing, Megan A. Mullins, K. Robin Yabroff, Joshua M. Liao

**Affiliations:** 1 Division of Hematology and Oncology, Department of Internal Medicine, University of Texas Southwestern Medical Center, Dallas; 2 Peter O’Donnell Jr. School of Public Health, University of Texas Southwestern Medical Center, Dallas; 3 Department of Medicine, University of Oklahoma Health Sciences Center, Oklahoma City; 4 Division of General Internal Medicine, Department of Internal Medicine, University of Texas Southwestern Medical Center, Dallas; 5 Program in Cancer Health Economics Research, Jonsson Comprehensive Cancer Center, and Department of Radiation Oncology, School of Medicine, University of California, Los Angeles; 6 Department of Internal Medicine, Sinai Hospital, Baltimore, Maryland; 7 Program on Policy Evaluation and Learning, Dallas, Texas; 8 Surveillance and Health Equity Science, American Cancer Society, Atlanta, Georgia

## Abstract

This cross-sectional study of national data on Part D and Medicare Advantage prescription drug plans compares utilization management and plan-level patient out-of-pocket costs for varenicline in smoking cessation treatment.

## Introduction

Smoking cessation is critical for promoting greater patient and population health.^1^ Varenicline, a nicotinic receptor partial agonist, is an effective smoking cessation medication.^2^ Despite its proven efficacy,^2^ varenicline use remains suboptimal, particularly among Medicare beneficiaries with a high burden of smoking-related diseases. Key barriers include utilization management (eg, prior authorization, quantity limits) and high out-of-pocket (OOP) costs.^3^ Meanwhile, Medicaid and private insurance beneficiaries often have zero cost-sharing for such therapy.

In the US, 80% of Medicare beneficiaries receive prescription drug benefits through Part D prescription drug plans (PDPs) or Medicare Advantage prescription drug plans (MAPDs).^4^ Given potential access and cost differences, we compared utilization management and plan-level patient OOP costs for varenicline between MAPDs and PDPs.

## Methods

In this cross-sectional study, we analyzed the Centers for Medicare and Medicaid Services 2023 Quarter 3 PDP and MAPD data.^5^ We examined utilization management strategies (prior authorization, quantity limit requirements) for 0.5 mg and 1 mg oral varenicline dosages, total cost, and estimated OOP costs for 12-week and 24-week treatment courses when filled at the health plan’s preferred retail pharmacy. To establish a standardized baseline for cross-plan comparisons, we estimated OOP costs under 2 scenarios: (1) before meeting the deductible and (2) having met the deductible and during the initial coverage phase. We used Wilcoxon and χ^2^ tests to compare costs and prevalence of management tools between PDPs and MAPDs. Analyses were conducted with SAS version 9.4 (SAS Institute Inc). A 2-sided *P* < .05 indicated statistical significance. This study followed the Strengthening the Reporting of Observational Studies in Epidemiology (STROBE) reporting guideline, and it was exempt from UTSW Human Research Protection Program review and informed consent as it was not human participant research.

## Results

**Figure 1.**
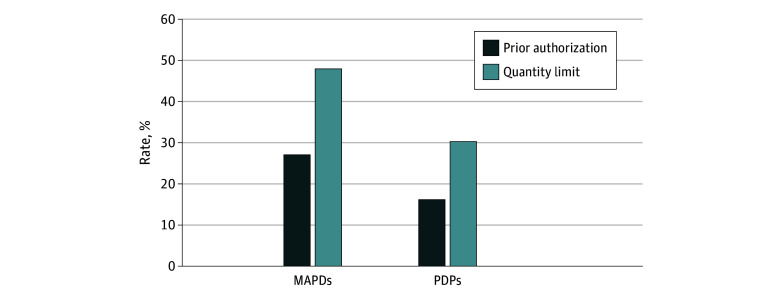
Prior Authorization and Quantity Limits Rates for Varenicline in Medicare Advantage Prescription Drug Plans (MAPDs) and Independent Part D Prescription Drug Plans (PDPs) Utilization management strategies did not differ by drug dosages in both MAPDs and PDPs. Prior authorization and quantity limits are identical for both 0.5 mg and 1 mg doses in MAPDs and PDPs. To concentrate on the plans most widely available to the public, we include Health Maintenance Organization (HMO), HMO-Point of Service, and Preferred Provider Organization plans and excluded employer-sponsored and Supplemental Need Plans (including those for dual-eligible individuals) as well as plans with fewer than 10 enrollees per Centers for Medicare & Medicaid Services plan enrollment in October 2023.

Among 813 PDPs and 3536 MAPDs, MAPDs had higher rates of prior authorization (27.1% vs 16.2%) and quantity limit requirements (48.0% vs 30.4%) compared with PDPs for both varenicline 0.5 mg and 1 mg dose. Prior authorization and quantity limits are identical for both doses in MAPDs and PDPs (Figure 1).

**Figure 2.**
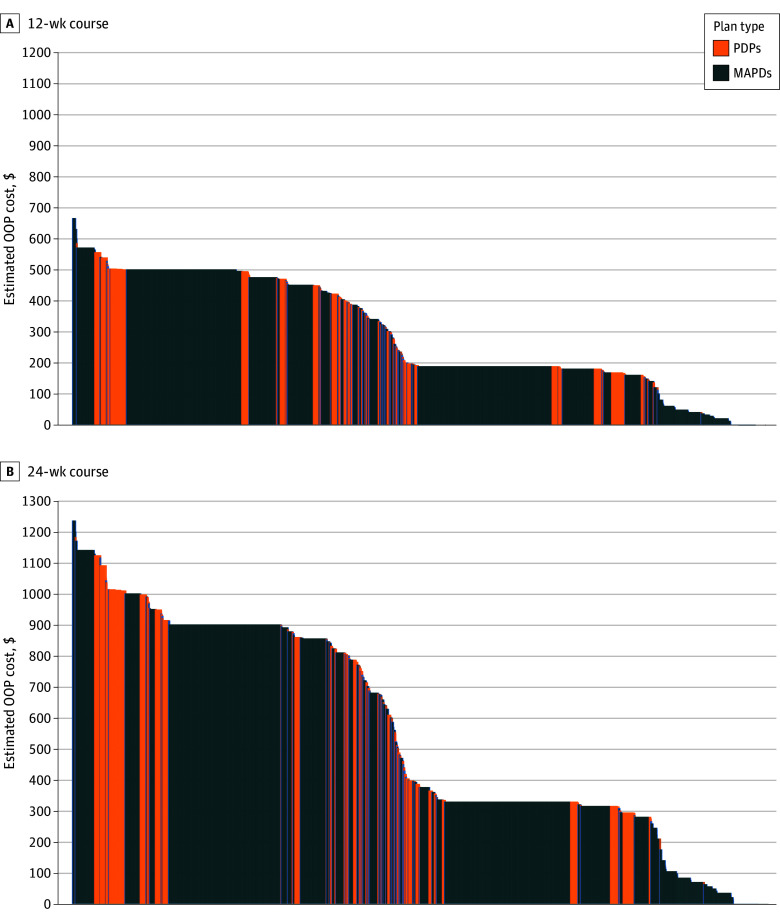
Out-of-Pocket (OOP) Costs of Varenicline Across Medicare Advantage Prescription Drug Plans (MAPDs) and Independent Part D Prescription Drug Plans (PDPs) To concentrate on the plans most widely available to the public, we included Health Maintenance Organizations (HMOs), HMO-Point of Service, and preferred provider organization plans and excluded employer-sponsored and Supplemental Need Plans (including those for dual-eligible individuals) as well as plans with fewer than 10 enrollees per Centers for Medicare & Medicaid Services plan enrollment in Oct 2023. To provide a consistent framework for comparing varenicline accessibility across different plans, OOP cost estimates assumed patients filled varenicline at their plan’s preferred retail pharmacy under 2 scenarios: (1) before meeting the deductible, and (2) having met their deductible, and during the initial coverage phase. We analyzed both 12-week and 24-week treatment courses to reflect common clinical prescribing patterns. The 12-week standard course was 0.5 mg daily for days 1-3, 0.5 mg twice daily for days 4-7, and 1 mg twice daily from day 8 to the end of treatment (a total 11 × 0.5 mg tablets, 154 × 1 mg tablets); the 24-weeks extended course included 0.5 mg daily for days 1-3, 0.5 mg twice daily for days 4-7, and 1 mg twice daily from day 8 to the end of treatment (11 × 0.5 mg tablets and 322 × 1 mg tablets).

Compared with PDPs, MAPDs had slightly lower median (IQR) total costs for 12-week ($1023 [$886-$1111] vs $1036 [$1003-$1063]) and 24-week courses ($2064 [$2024-$2246] vs $2092 [$2024-$2145]), with greater variation. Before meeting deductibles, MAPDs showed lower median (IQR) OOP costs than PDPs and greater variation (12-week, $340 [$180-$500] vs $605 [$552-$717]; 24-week, $555 [$315-$900] vs $1021 [$757-$1189]). When deductibles were met, MAPDs also had lower OOP costs with similar variations (12-week, $188 [$180-$495] vs $389 [$188-$493]; 24-week, $329 [$315-$900] vs $786 [$343-$996]) (Figure 2). Notably, 163 of 3536 MADP plans (4.6%) waived cost-sharing vs none of the 813 PDPs plans.

## Discussion

We found MAPDs imposed prior authorization and quantity limit for varenicline more frequently than PDPs. MAPDs had lower median total and OOP costs but showed greater variation in these costs compared with PDPs.

Heterogeneity in coverage policies and costs across plans warrants attention, especially as Medicare Advantage now covers 50% of beneficiaries. While MAPDs’ higher use of prior authorization and quantity limits raises concerns about administrative burden and barriers to smoking cessation treatment, lower median OOP costs and zero cost-sharing in some MAPDs may improve drug access. However, cost-sharing variability could lead to inequities in smoking cessation access.

Together, these findings suggest a need to enhance varenicline coverage alongside other preventive services, given the medication’s Grade A recommendation from the US Preventive Services Task Force.^6^ Ultimately, waiving patient cost-sharing and removing prior authorization for such high-value interventions across prescription drug benefits may promote use of varenicline and improved health outcomes through more smoking cessation.

This analysis is limited to plan-level data and may not capture patient outcome. Future research should examine how coverage differences influence varenicline use and cessation rates across regions with varying smoking burdens.

## Notes

Supplement.
Data Sharing Statement
