# Supplementary material for: Prior Authorization, Quantity Limits, and Costs for Varenicline in Medicare
Source: JAMA Netw Open. 2025 Mar 3;8(3):e250008. doi: 10.1001/jamanetworkopen.2025.0008 (PMC11877188; doi:10.1001/jamanetworkopen.2025.0008)
Supplement: Supplement. — Data Sharing Statement [file jamanetwopen-e250008-s001.pdf]

Data Sharing Statement

Data
Data available: Yes
Data types: Other (please specify)
Additional Information: Publically available data
How to access data: Publically available data
When available: With publication

Supporting Documents
Document types: None

Additional Information
Who can access the data: Publically available data
Types of analyses: Publically available data
Mechanisms of data availability: Publically available data
